# Global transmission of broad-host-range plasmids derived from the human gut microbiome

**DOI:** 10.1093/nar/gkad498

**Published:** 2023-06-07

**Authors:** Lili Yang, Guoqin Mai, Zheng Hu, Haokui Zhou, Lei Dai, Ziqing Deng, Yingfei Ma

**Affiliations:** CAS Key Laboratory of Quantitative Engineering Biology, Shenzhen Institute of Synthetic Biology, Shenzhen Institutes of Advanced Technology, Chinese Academy of Sciences, Shenzhen 518055, China; CAS Key Laboratory of Quantitative Engineering Biology, Shenzhen Institute of Synthetic Biology, Shenzhen Institutes of Advanced Technology, Chinese Academy of Sciences, Shenzhen 518055, China; CAS Key Laboratory of Quantitative Engineering Biology, Shenzhen Institute of Synthetic Biology, Shenzhen Institutes of Advanced Technology, Chinese Academy of Sciences, Shenzhen 518055, China; CAS Key Laboratory of Quantitative Engineering Biology, Shenzhen Institute of Synthetic Biology, Shenzhen Institutes of Advanced Technology, Chinese Academy of Sciences, Shenzhen 518055, China; CAS Key Laboratory of Quantitative Engineering Biology, Shenzhen Institute of Synthetic Biology, Shenzhen Institutes of Advanced Technology, Chinese Academy of Sciences, Shenzhen 518055, China; BGI-Shenzhen, Shenzhen 518083, China; BGI-Beijing, Beijing 102600, China; CAS Key Laboratory of Quantitative Engineering Biology, Shenzhen Institute of Synthetic Biology, Shenzhen Institutes of Advanced Technology, Chinese Academy of Sciences, Shenzhen 518055, China

## Abstract

Broad-host-range (BHR) plasmids in human gut bacteria are of considerable interest for their ability to mediate horizontal gene transfer (HGT) across large phylogenetic distance. However, the human gut plasmids, especially the BHR plasmids, remain largely unknown. Here, we identified the plasmids in the draft genomes of gut bacterial isolates from Chinese and American donors, resulting in 5372 plasmid-like clusters (PLCs), of which, 820 PLCs (comPLCs) were estimated with > 60% completeness genomes and only 155 (18.9%) were classified to known replicon types (*n* = 37). We observed that 175 comPLCs had a broad host range across distinct bacterial genera, of which, 71 were detected in at least two human populations of Chinese, American, Spanish, and Danish, and 13 were highly prevalent (>10%) in at least one human population. Haplotype analyses of two widespread PLCs demonstrated their spreading and evolutionary trajectory, suggesting frequent and recent exchanges of the BHR plasmids in environments. In conclusion, we obtained a large collection of plasmid sequences in human gut bacteria and demonstrated that a subset of the BHR plasmids can be transmitted globally, thus facilitating extensive HGT (e.g. antibiotic resistance genes) events. This study highlights the potential implications of the plasmids for global human health.

## Graphical Abstract

**Graphical Abstract:**
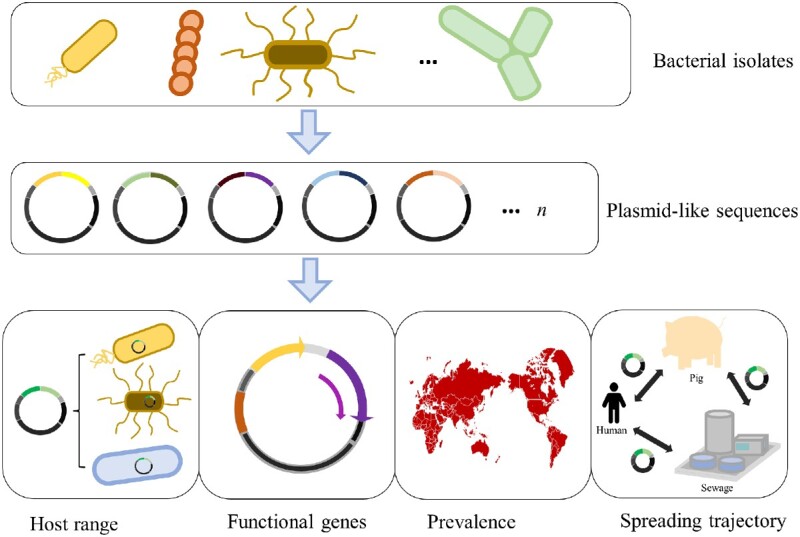

## INTRODUCTION

The human gut hosts a complex ecosystem comprising a multitude of bacterial species and thus is a hotspot for horizontal gene transfer (HGT) (1,2). Plasmids, which are a type of extrachromosomal mobile genetic elements of microbes, are considered one of the important mediators of HGT among microbes. Studies have demonstrated that some plasmids can spread fitness genes, e.g. salt tolerance genes (3) and antibiotic resistance genes (4), in the human gut microbiome. This suggests that the gut plasmidome (the overall human gut plasmid population) is indispensable and is expected to play important roles in HGT events in the human gut microbiome.

Broad host range (BHR) plasmids carrying fitness genes can transfer between bacteria across distantly phylogenetic taxa, thus facilitating the adaptation of their bacterial hosts to varying environments. For example, pB10, an IncP-1 type plasmid, has the ability to transfer between and replicate in nearly all species of the *Alpha-, Beta-* and *Gamma*-proteobacteria (5). These BHR plasmids were mainly observed in bacterial hosts isolated from the clinic or various environments. The BHR plasmids in the human gut are of considerable interest because they can disperse fitness genes in the microbiome, and their replicons can serve as a source for genetic vector construction that can be potentially applied in modulating the human gut microbiome. Some bioinformatic tools for identifying plasmid sequences in metagenomic sequencing data have been developed, including the machine learning tools, like PlasFlow (6), PlasForest (7) and PPR-meta (8), and assembly graph-based tools, like metaPlasmidSPADES (9), Recycler (10) and SCAPP (11). A study collected a large number of plasmid sequences (*n* = 92 492) from metagenomic datasets of different human body sites including the human gut by the bioinformatic methods (12). However, metagenomic data like this cannot provide accurate plasmid host information. This information can enable us to assess the extent to which the gut plasmid host range is broad and understand the effects of the transmission of the BHR plasmids in the human gut ecosystem on human health.

In this study, taking advantage of the draft genomes of human gut bacterial isolates generated in two culturomics-based studies of the human gut microbiome (13,14), we identified and characterized the plasmid-like sequences (PLSs) harbored by the isolates. We subsequently determined their accurate host range, persistence in the human gut, accessory genes, prevalence across various environments, and spreading and evolutionary trajectories. This resulted in a large collection of information on human gut BHR plasmids and showed their extensive global transmission. This characterization of the plasmids will thus greatly increase our understanding of the gut microbiome.

## MATERIALS AND METHODS

### Datasets used in this study

In total, 4983 draft genomes of the human gut bacterial isolates were used to identify the human gut bacterial plasmids from the studies (Supplementary Figure S1 and Supplementary Table S1) (13,14). These bacterial isolates were collected from the fecal samples of Chinese donors (*n* = 155) and American donors (*n* = 11). 1520 genomes of Chinese donors and 3463 genomes of American human donors could be downloaded. Metagenomic sequencing data, including human gut metagenomic sequencing datasets (15), environmental urban sewage metagenomic sequencing datasets (16), and pig gut bacterial metagenomic sequencing datasets (17), were used to investigate the prevalence of the identified plasmids (Table S2). The human gut metagenomic sequencing datasets included 368 Chinese samples, 139 American samples, 401 Danish samples, and 359 Spanish samples. The pig gut metagenomic sequencing datasets included 87 Chinese samples, 100 Danish samples and 100 French samples. The sewage water samples were from Africa (15 samples), Asia (9 samples), Europe (31 samples), the Middle East (3 samples), North America (14 samples), Oceania (3 samples) and South America (6 samples).

The complete bacterial genomes including bacterial plasmids (*n* = 19 073) and chromosomes (*n* = 22 311) were obtained from the NCBI database (ftp://ftp.ncbi.nlm.nih.gov/genomes/GENOME_REPORTS, date: 2020/05/26), and these sequences were managed as references for identifying plasmids by global similarity. The NCBI nonredundant (NR) gene dataset was obtained from the website ftp://ftp.ncbi.nlm.nih.gov/ (date: 2019/07/26).

### Bioinformatic workflow for identifying plasmid-like sequences (PLSs)

**Figure 1.**
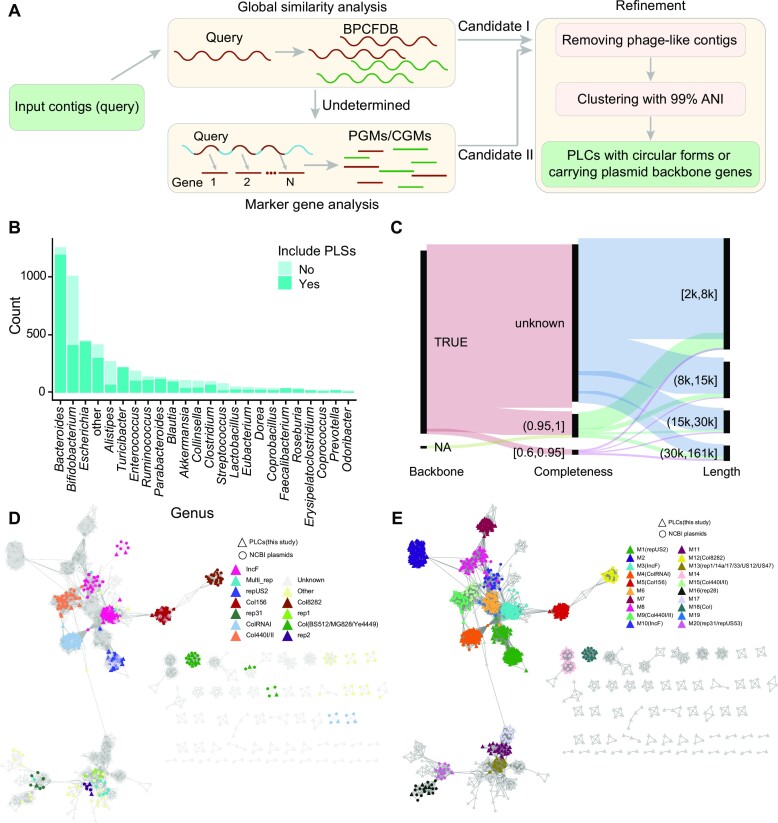
Identification and characterization of the identified PLSs from human gut bacterial isolates. (**A**) Bioinformatic workflow for the identification of PLSs from human gut bacterial isolates. BPCDB, bacterial plasmid and chromosome sequence fragment database. PGMs, plasmid-like gene markers. CGMs, chromosome-like gene markers. PLCs, plasmid-like clusters. Details in Materials and Methods. (**B**) Number of isolates from different bacterial genera with and without PLSs. ‘Other’ denotes unknown genera or genera with fewer than 20 isolates. (**C**) Overall summary of the gene content, completeness and lengths of these identified PLCs. The height of each bar is proportional to the number of PLCs in the category. (**D**) Network layout showed consistency with replicon typing. For each comPLC, its closest 3 NCBI plasmids with Mash distance <0.15, if existed, were included in this network. An edge represented a pair of sequences with distance < 0.15. The layout was ‘Prefuse Force Directed Layout (unweighted)’. The replicon types of comPLCs, detected by PlasmidFinder (25,30), were mapped on the network. E. The results of automatically detecting network typing groups (NTGs) by ‘clusterMaker’ (MCL cluster) (32). The 20 largest NTGs on the network were shown. The comparable replicon type of a NTG, if known, was shown after ‘::’.

We developed a workflow to identify PLSs (≥2 kb) from the draft genomes of the bacterial isolates. This workflow is summarized in Figure 1A.

First, in the draft genomes of the gut bacterial isolates, we identified candidate plasmid-like sequences (PLSs) (Candidate I) with global similarity to the NCBI known plasmid sequences and excluded the sequences with much global similarity to the NCBI known bacterial chromosomes (Figure 1A). To control the bias in sequence length between the draft genome sequences (often falling in 2–200 kb) and the reference sequences, we constructed BPCFDB (Bacterial Plasmid and Chromosome Sequence Fragment DataBase) as a reference (Supplementary Figure S2 A, see ‘Constructing BPCFDB’). The distance between each input contig and BPCDB was computed by the software Mash (*k* = 17) (18). A contig was assigned as a candidate PLS if it was aligned to more plasmid fragments than chromosome fragments with a distance <0.15. Those contigs that were not aligned to any fragments in BPCDB with a distance <0.15 were temporarily assigned as undetermined sequences.

Second, we identified candidate II PLSs from the ‘undetermined’ sequences according to plasmid gene markers for potentially novel plasmids (Figure 1A). A contig was assigned as PLS if it encoded at least one plasmid-like gene marker (PGM) but did not encode any chromosome-like gene marker (CGM) (Diamond, *e*-value < 0.001) (19). The PGMs and CGMs were selected from the Pfam genes by their frequency on bacterial chromosome and plasmid sequences (Supplementary Figure S2B and C, see ‘Selecting PGMs and CGMs’).

### Refinement for high-confidence PLSs and clustering

All the identified PLSs (candidates I + II) were examined to further remove phage-like contigs. This would also remove potential plasmid-phage elements as described by Rocha *et al.* (20). The contigs that encoded phage-like genes (including *terminase, holin, head, tail, portal* and *capsid*) were removed manually (Figure 1A).

The candidate PLSs were clustered into candidate plasmid-like clusters (PLCs) by the Python Scipy package with the function ‘cluster_hierarchical’ (linkage = ‘complete’, linkage_cutoff = 0.01) based on the distance matrix generated by Mash (*s* = 3000) (18). This resulted that pairwise PLSs had a Mash distance less than 0.01 (roughly equal to ANI 99%) within a candidate PLC. The protein-coding genes of PLSs were predicted by Prodigal (21). Plasmid backbone functional genes responsible for replication, mobilization, conjugation, segregation, and stabilization (keywords including ‘*rep*’, ‘*rop*’, ‘*primase*’, ‘*mob*’, ‘*mbp*’, ‘*relaxase*’, ‘*conj*’, ‘tra’, ‘trb’, ‘resolvase’ and ‘partition’) were detected manually in the NR annotation (Diamond (19), *E*-value 1e-10). We retained the candidate PLCs with any members having ∼100% completeness (see ‘Estimation the completeness of identified PLSs’) or encoding plasmid backbone genes as final PLCs due to their high confidence. This curated human gut plasmid collection includes 17 594 PLSs, which could be clustered into 5372 PLCs (Supplementary Table S3).

### Constructing BPCFDB

The NCBI bacterial chromosome and plasmid sequences were cut into 200 kb non-overlapping fragments. Most plasmids were less than 200 kb, which would not be cut. This aimed to mainly reduce the overestimation of distance calculated by Mash between 2–200 kb chromosome-like contigs in the queries and reference chromosomes. Pairwise distances among these fragments were calculated by Mash (*k* = 17). Then, these fragments were clustered by MCL (distance < 0.02) to reduce redundancy (22). The resulting representative fragments were collected in BPCFDB (Supplementary Figure S2 A).

### Selecting PGMs and CGMs

We selected PGMs and CGMs from Pfam genes according to their frequency on plasmids and chromosomes. To control the bias of computing Pfam gene frequency on complete plasmids/chromosomes for identifying plasmids in the draft genomes, we constructed a dataset that included 16 000 plasmid fragments and 160 000 chromosome fragments 2–200 kb in length (Supplementary Figure S2B). They were randomly cut from NCBI bacterial plasmids and chromosomes. The protein sequences encoded by these plasmid/chromosome fragments were compared against the protein sequences of Pfam database using Diamond (*e*-value < 0.001) (19). We then calculated the frequency of each Pfam gene on the plasmid sequences (FP) and the frequency of each Pfam gene on the chromosome sequences (FC). The genes that occurred with low frequency on both chromosomes (FC < 0.2%) and plasmids (FP < 0.2%) were removed. We visualized the distribution of the fragments encoding tentative PGMs but not encoding tentative CGMs based on FP/FC values (Figure S2C). When the number of tentative CGMs (with bottom FP/FC values) increases and the number of tentative PGMs (with top FP/FC values) decreases, it can increase the accuracy for plasmid prediction but decrease the recall rate, as shown. We manually selected the 212 Pfam genes with FP/FC >7 as PGMs and the Pfam genes with the bottom 1400 FP/FC values as CGMs to obtain high precision and a moderate recall rate (Supplementary Table S4).

### Assessing the performance of the workflow

To assess the performance of the workflow in identifying the candidate PLSs (candidate I + II)) from the draft genomes of the human gut bacterial isolates, we used the NCBI sequences to mock the draft genome sequences. The mimic sequences were randomly cut from 20% NCBI bacterial plasmid and 20% chromosome sequences and then were resampled by R script for even length distribution. The mimic data included 5000 plasmid fragments and 5000 chromosome fragments 2–200 kb in length (Supplementary Table S5). The remaining 80% of NCBI chromosome and plasmid sequences were constructed as 80%-BPCFDB in the same way as BPCFDB. We predicted 3520 PLSs by comparing the mimic contigs with the 80%-BPCFDB and 501 PLSs by CGMs and PGMs (Supplementary Table S5). Only 23 predicted PLSs were false positives. In total, the workflow had 99.4% precision and 80.0% recall. The precision was roughly maintained even using different lengths of mimic contigs (Supplementary Figure S2 D). The recall slightly decreased as the length of the mimic contigs increased but was always >70%.

### Benchmarking

To further assess the workflow in identifying plasmids in human gut bacterial isolates, we run our workflow, PPR-Meta (8), and PlasForest (23) on a subset of human gut bacterial isolates. For bacterial genera having more than 3 isolates, we randomly selected three isolates. Combining the selected isolates and all isolates from genera that have no more than three isolates, we obtained 211 isolates covering > 85 bacterial genera for the test (Supplementary Table S6). PPR-Meta predicted 1783 PLSs, including 475 high-confidence PLSs (having plasmid backbone genes or with ∼100% completeness, see ‘Estimation the completeness of identified PLSs’). PlasForest predicted 123 PLSs, including 36 high-confidence PLSs. Our workflow predicted 838 PLSs, including 561 high-confidence PLSs (Supplementary Table S6). Therefore, our workflow obtained the largest set of high-confidence PLSs and the largest ratio of high-confidence PLSs to all predicted PLSs compared with PPR-Meta and PlasForest. Some sequences that were determined to be PLSs by PlasForest or PPR-Meta were identified as chromosome sequences by our workflow. Our workflow excluded them from PLSs due to their global similarity with known chromosomes or chromosome-like genes, which could further reduce false positives.

In particular, we ran PPR-meta and PlasForest on the top 10 PLCs with a high number of members (Supplementary Table S6). The 10 PLCs included 2730 PLSs according to our workflow. Then, 1733 PLSs were obtained by PPR-meta, and 385 PLSs were obtained by PlasForest. This finding indicated that PlasForest and PPR-meta missed many high-confidence PLSs again. In addition, PlasForest and PPR-meta cannot obtain coherent results for all members in a PLC. For example, PlasForest and PPR-meta recalled 5 and 326 members of Clstr_599, respectively, while Clstr_599 included 384 members obtained by our workflow. Clstr_599 had a Mash distance <0.001 with NCBI known plasmids. Due to the pairwise ANI >99% for all members in a PLC and the typical characteristics of plasmids, all members should be assigned as plasmids but not just part of these members.

### Estimation of the completeness of the identified PLSs

We determined ∼100% complete sequences of PLCs through two steps (Supplementary Figure S3 A): (i) the longest PLS within a given PLC was selected and was detected with direct terminal repeats (DTRs) using EMBOSS merger (24). The PLS with DTRs (length ≥ 10 bp and mismatch ≤ 1) was considered for next step; (ii) for a PLS, we removed the ending DTR and mapped the sequencing reads of the corresponding genome to the two ends (300 bp) of the PLS using BLAST (25). The reads were considered hits if each had > 90% coverage and each mapped to at least 20 bp of the PLS ends except the DTR region. A PLS was considered with ∼100% completeness if there were paired reads mapped to two ends of the PLS. A similar method has been used for estimating circular plasmids before (26). There were 509 PLCs passed the first step. Finally, 483 PLCs with ∼100% completeness were obtained.

We estimated the completeness for each of the remaining PLSs based on their assembly graph in a way similar to the method for estimating ∼100% completeness (Supplementary Figure S3B). Only those PLSs with a circular path in an assembly graph were considered. Detailed steps were as follows:

- Obtaining assembly graph. The sequencing reads of each genome of the bacterial isolates were trimmed by fastp (-q 20 -u 20 -l 40 -5 -3) (27) and were reassembled using SPAdes (28). This generated a file, i.e. ‘*assembly.fastg*’, with the information of assembly graph for each bacterial isolate, including nodes representing assembled fragments and the linkages among nodes. The absolute abundance for each node was given in the file of ‘*assembly.fastg’*.
- Determining the shortest circular path for each PLS. Each PLS was compared for the best hit using Blastn (25) in *assembly.fastg*. The shortest circular path for each PLS was determined by the python package NetworkX (29) from *assembly.fastg*. A PLS with the circular path was selected. The path for each PLS often had multiple nodes in order (Supplementary Figure S3B). The copy number of each node in the shortest path was then estimated using *C* = (*A* – min(*A*_p_, *A*_f_))/*A*_base_. *A* is the absolute abundance of the node itself. *A*_p_ and *A*_f_ are the sum of the absolute abundance of the adjoining nodes on its two ends, respectively, and the adjoining nodes are not in the path. *A*_base_ is the absolute abundance of the PLS in *assembly.fastg*.*C* > 1.8 was seen as multiple copy number for a node in a path, except for nodes in a path with two opposite directions, which was seen with multiple copy numbers when *C* > 2.8.
- Validating the shortest path using paired reads. The paired reads were mapped to all nodes for a circular path by BLAST (25). The reads with > 90% coverage in the path were considered. Based on the mapping results, we checked whether all adjoining nodes in the shortest path for each PLS were mapped by paired reads to validate the path. For a node with multiple copy numbers, only when there were paired reads mapped to its upstream and downstream adjoining nodes, it was not considered as the signal for skipping integrative elements in a shortest circular path and taken for further analysis (Supplementary Figure S3 B).
- Computing the completeness. For each PLS, the completeness was estimated using *L*_PLS_/(*L*_PLS_ + *L*_G_). *L*_PLS_ is the length of PLS. *L*_G_ is the sum of the length of all nodes in the shortest path.

The PLSs with completeness >60% were assigned as comPLSs and the PLCs with at least one comPLSs were assigned as comPLCs. This resulted 7256 comPLSs that belonged to 820 comPLCs, including 483 PLCs with ∼100% completeness (Supplementary Table S7).

### Plasmid typing based on network analysis and using PlasmidFinder

Replicon typing was conducted for the comPLCs using PlasmidFinder (25,30) (Supplementary Table S8). We also conducted plasmid typing for the comPLCs and their similar NCBI plasmids using network. Firstly, top 3 plasmids with Mash distance <0.15 for each comPLC were selected from the NCBI database if existed. Then the pairwise Mash distance of the chosen NCBI plasmids and the comPLCs were recalculated. The network was visualized using Cytoscape (31) with the layout ‘Prefuse Force Directed Layout (unweighted)’. The input table included network edges representing pairs of sequences with distance <0.15. Thus, the singleton comPLC without similar sequences with distance <0.15 was not included. This resulted in a network including 745 comPLCs (Supplementary Table S8). The algorithm ‘clusterMaker’ (MCL cluster) (32) in Cytoscape was used to classify network typing groups (NTGs). The consistent results between network typing and replicon typing showed the feasibility of the network typing method for novel plasmids.

### Detection of the gene contents, host range, persistence and prevalence of PLCs

CONJscan (33) was used to determine conjugative PLSs. A PLC was considered as a conjugative PLC if any member PLS was conjugative. Gene annotation against the NCBI nonredundant database was used to determine mobilizable PLSs if they encoded MOB genes by keywords search and manual check. A PLC was considered as a mobilizable PLC if any member PLS was mobilizable and no member PLS was conjugative. Antibiotic resistance genes (ARGs) were detected by CARD’s Resistance Gene Identifier (34). Virulence genes in the PLSs were annotated in the virulence gene database (VFDB) using Diamond (*E*-value = 1e-10, identity > 80%, coverage > 80%) (35). Carbohydrate-active enzymes (CAZymes) were annotated by dbcan2 (36) against the CAZy database (37).

The host range of a PLC was determined according to the bacterial isolates from which its member PLSs were derived. The taxonomic IDs of the bacterial isolates were downloaded by Bio.Entrez in Python (38). The network of the bacterial genera was connected by the shared PLCs and visualized by Cytoscape (31). The phylogenetic tree of the gut bacterial isolates was constructed by PhyloPhlan (39) using their genome sequences without the identified PLSs. Visualization of the phylogenetic tree was performed using ggtree (40). The persistence of each BHR PLC in American_am gut was detected if any members of the PLC were detected in any isolates across different time points.

Metagenomic datasets of various human populations and environmental niches were used to detect the presence of PLCs. The human gut metagenomic contigs were downloaded directly. The cleaned metagenomic reads of the pig gut could be downloaded directly. Reads of sewage metagenomes were trimmed by fastp (-q 20 -u 20 -l 40 -5 -3) (27). The cleaned reads were assembled into contigs using Megahit (41). These assembled contigs (≥2k bp) were compared to the identified member PLSs of PLCs using Mash. One PLC was considered present in a sample if any members of the PLC had a pairwise Mash distance less than 0.01 with any contigs of the sample. The similarity of ARGs was obtained by Usearch (42).

### Haplotype analysis of the representative widespread PLCs

We constructed haplotype networks to explore the evolutionary and spreading patterns. Only members with DTRs (length ≥ 10 bp, mismatch = 0) were considered. The overlapping regions at two ends for each contig were removed. Then, the sequences were reformatted to use the same beginning point and in the same strand by Python script. This could avoid the influence of cyclically permuted sequences and sequence directions on alignments. Then, MUSCLE (43) was used to perform sequence alignments. Taking the members with median length as references, the members having long indels (≥2 bp) in the alignment were removed.

DnaSP (44) was used to generate haplotype data from the alignment. The single-base indels were also considered into haplotypes. This ensured that the number of different bases between the two haplotypes reflected all differences in the complete sequences they represented. Then, PopART (45) was used to construct the median joining network for haplotypes.

## RESULTS

### Diverse plasmids derived from human gut bacterial isolates

To identify plasmid-like sequences (PLSs), 4983 draft genomes of human gut bacterial isolates were used (13,14), with an average of 74.93 contig sequences (>2 kbp) per isolate. Among them, 1520 isolates were cultured from 155 healthy Chinese human donors, and 3463 were cultured from 11 healthy American human donors (Supplementary Table S1, Figure S1). These isolates belonged to > 85 genera (85 known genera and other unknown genera), and 23 genera had more than 20 isolates. *Bacteroides* (*n* = 1265) and *Bifidobacterium* (*n* = 1009) constituted the largest portion of these isolates at the genus level. Notably, of the American isolates, 1293 bacterial isolates belonging to 25 genera were from an individual American people (am, called ‘American_am’ hereafter) (14).

We developed a workflow to identify PLSs from the draft genomes of these bacterial isolates (Figure 1A, Materials and Methods). First, we identified candidate PLSs with high global similarity to the plasmid sequences in the bacterial plasmid and chromosome sequence fragment database (BPCFDB, see Materials and Methods for ‘BPCFDB’ and Supplementary Figure S2) using Mash (<0.15) (18) in each draft genome, resulting in 18 871 candidate PLSs (candidate I). The sequences with high similarity with chromosome sequences were discarded. Second, for the sequences without hits in BPCFDB, those encoding plasmid-like gene markers (PGMs, see Materials and Methods and Supplementary Figure S2B and C) were assigned to candidate PLSs (candidate II), resulting in 8772 candidate PLSs. The sequences encoding chromosome-like gene markers (CGMs, see Materials and Methods) were discarded. The performance of this workflow was validated using a mimic sequence dataset, showing that the precision in identifying plasmids reached 99.4% (Supplementary Figure S2D, Table S5). Benchmarking on a subset of contigs of human gut bacterial isolates showed that the workflow can recall more high-confidence PLSs (with plasmid backbone genes or a circular form) than PlasForest (23) and PPR-Meta (8) (see Materials and Methods, Supplementary Table S6).

Finally, we removed the phage-like sequences and retained the 17 594 high-confidence PLSs (Supplementary Table S3). The PLSs were obtained from 70.1% (3492/4983) of the isolates belonging to >72 genera (Supplementary Table S3), of which 3574 PLSs were in the bacterial isolates of more than 55 genera from Chinese donors and 14 020 PLSs were in the isolates of more than 42 genera from American donors. More than 95% of the isolates of *Faecalibacterium* (40/40), *Turicibacter* (220/224), and *Escherichia* (442/453) were shown to harbor PLSs (Figure 1B). We used pairwise Mash distances 0.01 (1 – average nucleotide identity (ANI)) (see Discussion) to group these PLSs, resulting in 5372 plasmid-like clusters (PLCs) (Supplementary Table S3). Of the total PLCs, 1801 were obtained by global similarity analysis with BPCFDB, 3591 were obtained by PGM and CGM analysis. The plasmid backbone genes responsible for replication, mobilization, conjugation, or stabilization were detected in the PLSs of 5301 PLCs (98.7%) (Supplementary Table S3, Figure 1C). The length of the PLCs ranged from 2 kb to 161 kb, and most (79.5%) PLCs were less than 15 kb in size (Figure 1C). We assessed the completeness for these PLSs (see Materials and Methods, Supplementary Figure S3), resulting in 7256 PLSs with >60% completeness (comPLSs) (Supplementary Table S7). These comPLSs belonged to 820 PLCs (comPLCs) (Supplementary Table S7), including 483 PLCs with ∼100% completeness. Of the 820 comPLCs, 492 were identified as mobilizable plasmids and 74 were conjugative plasmids (Supplementary Table S7).

The replicon typing of 820 comPLCs was obtained using PlasmidFinder (25,30). Only 155 (18.9%) were assigned to known replicon types (*n* = 37), mainly Col156 (*n* = 32), IncF (*n* = 30), ColRNAI (*n* = 28), repUS2 (*n* = 28), etc. (Supplementary Table S8). Other types including IncB/O/K/Z, IncH1B, IncR, IncX and CoII were also detected (Supplementary Table S8). The network-based method has been suggested for large-scale plasmid classification in previous studies (46,47). We constructed whole sequence similarity network of the comPLCs with their highly similar NCBI plasmid sequences, which was visualized by Cytoscape with the replicon typing information (see Materials and Methods, Figure 1D). Only pairs of sequences with Mash distance <0.15 were considered for the network. Thus, 745 comPLCs were included in this network (Figure 1D). Then the algorithm ‘clusterMaker’(32) was used to classify the sequences into 103 network typing groups (NTGs) (Figure 1E). The known replicon types obtained by PlasmidFinder were largely consistent with the results of the NTGs (Figure 1D and E), similar with previous studies using network for plasmid classification(46,47). In addition, we found 1448 isolates harbored 2–7 NTGs (Supplementary Table S8), revealing the compatible plasmids in their hosts. Taking top 20 largest NTGs for example, which contained 577 of 820 PLCs (70.4%), 12 of the 20 NTGs were comparable to the known replicon types, like M1 to repUS2, M3 to IncF and M4 to ColRNAI. The repUS2 plasmids were found in human gut microbiome before (48). The IncF plasmids, i.e. M3 and M10, were prevalent in clinic environments (49,50). The conjugative plasmids (*n* = 74) were mainly in M6 (*n* = 22), M3::IncF (*n* = 14) and M7 (*n* = 13). The mobilizable plasmids (*n* = 492) were mainly in M5::Col156 (*n* = 27), M21 (*n* = 12), M6 (*n* = 9). Those PLCs that were unassigned to known replicon types formed multiple (*n* = 74) NTGs, likely representing the plasmids with novel replicon types (Supplementary Table S8). The genetic organization of the genomes of M1–M6 representative comPLCs were shown (Supplementary Figure S4). Overall, the network-based classification showed the novelty and diversity of our identified PLCs in the human gut microbiome.

### Human gut plasmids across phylogenetically and geographically distant bacterial hosts

According to the host bacteria of the member PLSs for each PLC, we determined the host range of the human gut plasmids. In this analysis, we excluded the bacterial hosts assigned to unknown genera. Among all the PLCs, 2546 were found in Chinese donors, 2944 in American donors, and 117 in both Chinese and American donors. Remarkably, 402 PLCs (including 7794 PLSs) had a broad host range, as they were detected in the hosts across at least two genera (Supplementary Table S3, see Discussion), of which, 53 were detected in the isolates from both Chinese and American donors. The sharing network of these PLCs implied the pattern of the extensive exchanges of some genes (e.g. ARGs) likely mediated by these plasmids across 55 bacterial genera and even distinct phyla in the human gut (Supplementary Figure S5).

**Figure 2.**
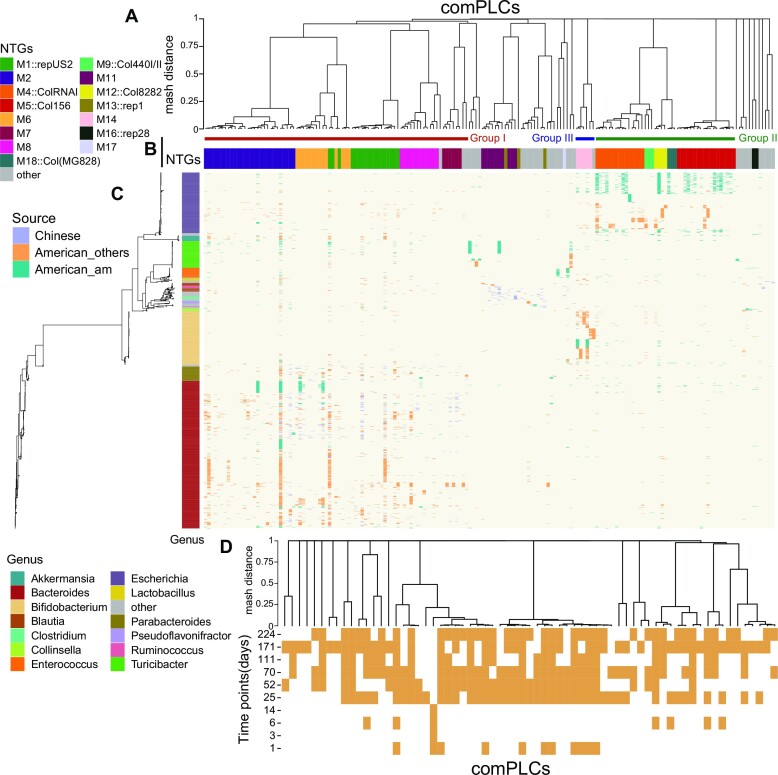
Distribution and persistence of human gut plasmids across distinct genera and geographically distant locations. 175 BHR comPLCs were selected for visualization. (**A**) The tree showing sequence diversity among PLCs. It was constructed according to the Mash distances between PLCs. (**B**) The network typing groups (NTGs) of comPLCs. The 20 largest NTGs were shown if they included any BHR comPLCs. The host genera *Bacteroides*, *Escherichia*, and *Bifidobacterium* most frequently occurred to these BHR plasmids, as shown in group I, II and III. (**C**) Distribution of PLCs in the bacterial isolates. The phylogenetic tree of bacterial hosts was constructed by PhyloPhlan (39) using the draft genomes excluding the identified PLSs. The heatmap shows the presence of each PLC in the bacterial hosts. The genera with more than 10 isolates here were shown. ‘Source’ denotes that the isolates were collected from the samples of American, Chinese, and the American individual (am). (**D**) Persistence of PLCs in the isolates sampled across different time points (days) from the American individual (am). Each column represents a PLC that was detected in the strains from the individual American_am across multiple phylogenetically distinct genera. The presence of a PLC was assigned if at least one member PLS of the PLC was detected in the isolates sampled at the time point.

We then focused on the 175 comPLCs (including 6590 PLSs) of 402 BHR PLCs, belonging to more than 33 NTGs. There were 27 of the 175 comPLCs presenting in the bacterial hosts from both Chinese and American donors. The hierarchical tree based on the Mash distance further showed the diversity of these PLCs in sequence (Figure 2A). The diversity was kept even only considering the cross-orders comPLCs (*n* = 135) (Supplementary Figure S6). The host genera *Bacteroides*, *Escherichia*, and *Bifidobacterium* most frequently occurred to these BHR plasmids, as shown in group I, II and III (Figure 2B and C). The PLCs of group I were detected in the genus *Bacteroides* (72.3%, 959/1291) more frequently than in other genera. They were clustered in eight NTGs, including the top two M1::repUS2 (comPLCs, *n* = 18) and M2 (comPLCs, *n* = 28). Six PLCs in group I were presented in the hosts across more than 10 genera, including 3 PLCs of the M1::repUS2 NTG (Clstr_981, 16 genera; Clstr_599, 13 genera; Clstr_388, 13 genera) and 3 of the M2 NTG (Clstr_417, 24 genera; Clstr_875, 11 genera; Clstr_424, 15 genera). Notably, Clstr_417 had the largest number (*n* = 730) of member PLSs (Supplementary Table S3), spreading among the isolates of 24 genera across 5 phyla, including Firmicutes, Actinobacteria, Proteobacteria, Verrucomicrobia and Bacteroidetes. The isolates including Clstr_417 were collected from Chinese donors (*n* = 27) and American donors (*n* = 11) (Supplementary Table S3). The PLCs of group II mainly (80.1%, 381/471) were hosted in *Escherichia*. The plasmids derived from *Escherichia* have been well studied. This may explain that all 5 NTGs in Group II were all corresponded to well-known replicon types including M4::ColRNAI, M5::Col156, M9::Col440I/II, M12::Col8282 and M18::Col. Group III had two NTGs including M14 and M78. The majority of the PLSs within this group (88.0%, 314/357) were present in the isolates belonging to *Bifidobacterium*. The PLCs, not belonging to group I, II and III, included more than 18 NTGs and were hosted in distinct bacterial genera (e.g.*Akkermansia, Blautia, Clostridium, Coprococcus, Turicibacter*) (Figure 2C). Of the 20 largest NTGs, we found that the PLCs detected in M3::IncF, M10::IncF, M15::Col440I/II, M19 and M20::rep31/repUS53 were limited to a specific genus. The IncP, IncN and IncW plasmids, widely known as BHR plasmids before, were not found in our comPLCs. The only IncQ plasmid in this study, clstr_7695, was only detected in the isolates of *Enterobacter* (Supplementary Table S3).

We then focused on comPLCs (*n* = 67) with broad host range that were derived from the individual American_am to assess the within-person persistence of the plasmids in gut microbiome (Figure 2C and D). The plasmid-derived isolates from American_am were longitudinally sampled over 224 days. We detected the presence of 67 comPLCs in the isolates sampled at each sampling time point (Figure 2D). Of these comPLCs, 53 (79.1%) PLCs can persist in the microbiome for at least 50 days, and 41 (61.2%) PLCs can persist even for at least 150 days. The long-term persistence of plasmids in the human gut microbiome might ensure the widespread transmission of human gut plasmids across distinct bacterial strains.

### Accessory functional genes encoded by human gut plasmids

**Figure 3.**
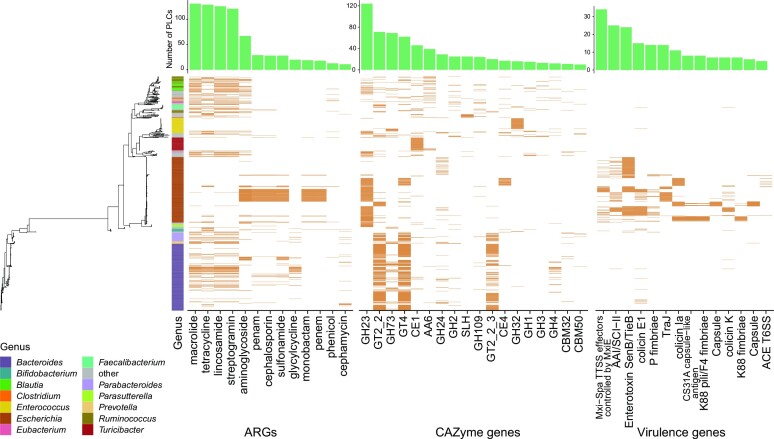
Distribution of accessory genes in the PLSs of PLCs. ARGs, antibiotic resistance genes; CAZyme, carbohydrate-active enzymes. These genes were annotated in the CARD (34), VFDB (35) and CAZy (37) databases. The number of PLCs that harbored the corresponding genes is shown. According to the number of PLCs involved in each type of gene, only the top 13 ARGs, top 18 CAZymes, and top 14 virulence genes are shown.

Three types of accessory genes responsible for antibiotic resistance, virulence, and carbohydrate-active enzymes were frequently detected in the PLSs (Supplementary Table S9 and Figure 3). A variety of antibiotic resistance genes (ARGs) were identified in the PLSs of 339 (6.3%) PLCs. The member PLSs of some PLCs carried multiple drug resistance genes (up to 7, including Clstr_3026, Clstr_4982, Clstr_7911, and Clstr_7913) (Figure 3 and Supplementary Table S9). Notably, the ARGs for aminoglycoside, cephalosporin, sulfonamide, monobactam, and penem antibiotics were mostly located on the PLSs of *E. coli*, and ARGs for macrolide, tetracycline, lincosamide, and streptogramin antibiotics were mainly located on the PLSs of bacterial taxa other than *E. coli* (across up to 19 genera) (Figure 3). The genes conferring resistance to macrolides, tetracycline, lincosamide and streptogramin occurred most frequently on the PLSs of 131, 129, 126 and 121 PLCs, respectively. This observation indicates that in the human gut microbiome, both potential pathogens (e.g.*E. coli*) and commensal bacteria (e.g.*Bacteroides*) are likely sources of ARGs.

We identified 162 families of carbohydrate-active enzymes (CAZymes) on the PLSs of 604 PLCs (11.2%). The types of enzymes encoded by the plasmids had host specificity at the bacterial taxon level to some extent, i.e. the genes encoding the enzymes GH23 (in 124 PLCs) were detected at the highest frequency in the PLSs of *E. coli*, while those encoding GT2_Glycos_transf_2 (in 71 PLCs), GH73 (in 69 PLCs) and GT2_Glyco_trans_2_3 were most frequently detected in the PLSs of *Bacteroides*. GH23 is a type of lysozyme, and the other 4 types of enzymes are involved in bacterial capsule biosynthesis.

In total, 115 distinct virulence genes were detected in 1040 PLSs of 143 PLCs (2.7%). A great majority (95.8%, 996/1040) of the PLSs carrying these virulence genes were detected in *E. coli* hosts in both the American and Chinese human isolates (Figure 3 and Supplementary Table S9). The genes encoding enterotoxin (in 23 PLCs) and colicin (including E1, la, lb, K and colicin-like Usp, detected in 14 PLCs) were the most frequently observed virulence genes in these plasmid sequences. Few PLSs identified from the isolates other than *E. coli* carried the colicin E1 gene from the isolates of *Bacteroides* (*n* = 8), *Faecalibacterium* (*n* = 2) and *Bifidobacterium* (*n* = 5).

Of the comPLCs (*n* = 820), the members of 52, 29, 29 comPLCs were detected with ARGs, virulence genes and CAZyme genes (Supplementary Table S9). They were present in 21, 6, 28 NTGs separately. We found that the BHR comPLCs encoded CAZyme genes with significantly lower frequency than other comPLCs (Fisher's Test, *P* < 0.01) but without significant difference for virulence genes and ARGs (Chi-square test, *P* > 0.5). Of the 175 BHR comPLCs, only 9 were identified as conjugative plasmids (all >40 kb) and 130 were mobilizable plasmids (averaged ∼10.3 kb) (Supplementary Table S7). The BHR comPLCs had more mobilizable plasmids (hi-square test, *P* < 0.05) and less conjugative plasmids (chi-square test, *P* = 0.06) than other comPLCs. In the BHR comPLCs, the conjugative plasmids spread across a significantly smaller number of genera than mobilizable plasmids (Wilcoxon test, *P* < 0.05). Analysis indicated that the mobilizable plasmids were detected with a broader host range than the conjugative plasmids. The accessory genes including VFs, CAZyme genes and ARGs occurred to the conjugative plasmids (9/74, 39/74, 10/74) with a higher frequency than to the mobilizable plasmids (12/492, 25/492, 26/492) (chi-square test, all *P* < 0.05). This might be due to the fact that the conjugative plasmids often have a large genome that can carry more genes.

### Human gut plasmids widely spreading in various human populations and environments

To determine the extent to which human gut plasmids spread in various environments, we collected metagenomic datasets for human gut samples (*n* = 1267) (15), pig gut samples (*n* = 287) (17) and sewage samples (*n* = 81) (16) from different locations (Table S2) to examine the presence of these plasmids in these distinct niches. All the sequences of the PLCs were compared to the assembled metagenomes, and one PLC was considered to be present in one sample if any members of the PLC had pairwise Mash values less than 0.01 with any contigs of the metagenomes.

**Figure 4.**
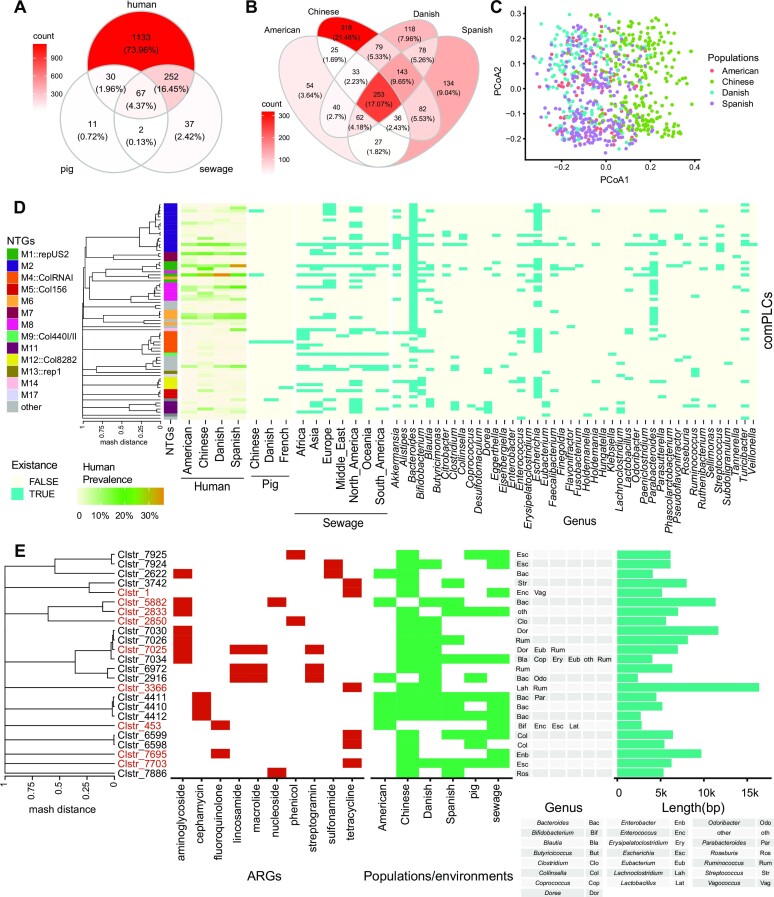
Prevalence of PLCs in different ecological niches and human populations. (**A**) Number of PLCs shared between different ecological niches, including the human gut, the pig gut, and sewage water. The presence of a PLC in a niche was indicated if at least one member of the PLC was detected in the samples of the niche. (**B**) Number of PLCs shared between different human populations. The presence of a PLC in a population indicated whether at least one member of the PLC was detected in the samples of the population (including Chinese, American, Danish, and Spanish samples). (**C**) PCoA of the identified PLCs present in different human populations. The PCoA was based on the presence/absence (1/0) of PLCs in each sample. Samples with <10 PLCs detected were excluded. The PLCs present in less than 10 samples were excluded. PCoA was conducted by the Vegan package in R. (**D**) 71 cross-genera PLCs occurring in at least two human populations. The prevalence of a PLC in one population was calculated by the number of samples with the PLC divided by the total number of samples in the population. (**E**) Distribution and hosts of the highly identical ARGs encoded by the PLCs shared between different environments. The colored names denote that the members of those comPLCs.

In total, 1531 PLCs were detected in at least one of these three types of niches, and 67 PLCs (4.4%) were detected concurrently across all three types of environmental niches (Figure 4A and Supplementary Table S10). In particular, 1482 PLCs were detected in the metagenomic samples of at least one of the human populations, namely, the Chinese, American, Spanish, and Danish populations. 253 PLCs were shared across all these populations and 858 PLCs were detected in at least two human populations (Figure 4B and Supplementary Table S10). Remarkably, principal coordinate analysis (PCoA) revealed that the composition of the human gut plasmidome was significantly different between the Chinese and Western populations (PERMANOVA, *P*-value = 0.001, Figure 4C). This suggested that living habits can likely affect the composition of the gut plasmidome.

Of the 175 BHR comPLCs inferred from the data of bacterial isolates, 71 (40.6%) were detected in at least two human populations here (Figure 4D, Supplementary Table S10). The 71 comPLCs belonged to 21 network types. There were 13 of the 71 comPLCs, belonging to 8 NTGs, showing high prevalence (>10%) in at least one human population. For example, the Clstr_599 (M1::repUS2), clstr_388 (M1::repUS2) and clstr_417 (M2), showing > 10% prevalence in at least one human population, had hosts across 13, 14 and 25 genera, respectively (Figure 4D, Supplementary Table S10). The Clstr_599 had the highest prevalence with 16.5%, 27.7%, 36.2%, 20.1% in American, Chinese, Danish, Spanish populations, separately. In addition, these comPLCs also showed global distribution in sewage, and Clstr_599 was also detected in the pig gut microbiome. This observation demonstrated that a subset of human gut plasmids with an extraordinarily broad host range can spread widely among various distinct environments.

These plasmids are so ubiquitous in various environments that they presumably are vectors that can mediate the horizontal transfer of accessory genes (*e.g*. ARGs) in the environment. We found that 24 PLCs, each of which including members encoding nearly identical ARGs (>99% identity), were presented in at least two human populations or environmental niches (Figure 4E). Besides, 7 of the 24 PLCs were determined from the bacterial isolates across different genera. For example, Clstr_7034, hosted in 6 bacterial genera carrying the genes for resistance to aminoglycoside antibiotics, was detected in the microbiome of the Chinese, Danish, and Spanish gut, the pig gut, and the sewage niche. Five PLCs carried genes with resistance to multiple types of antibiotics. For example, the Clstr_5882, encoding the ARGs for aminoglycoside and nucleoside antibiotics, was detected in the gut microbiome of American, Danish, and Spanish. Cosmopolitan bacteria with extensive environmental adaptability, e.g. *E. coli*, harboring these plasmids, might greatly contribute to the dissemination of ARGs as well as other fitness genes in various environments.

### Evolutionary and spreading trajectory of widespread PLCs

To trace the evolutionary and spreading trajectory of these widespread plasmids across different environmental reservoirs, we constructed haplotype networks for the member PLSs of the PLC Clstr_599. The members of this PLC (M1 NTG) were detected in the bacterial isolates across 5 phyla and showed the highest prevalence in various environments detected by metagenomic analysis among the identified PLCs. Only PLS members with single-base polymorphisms/indels were taken into consideration when generating haplotypes. Therefore, PLSs belonging to a haplotype shared 100% identity.

**Figure 5.**
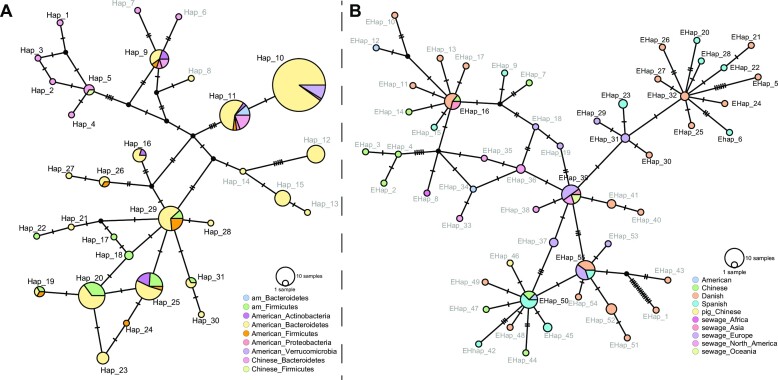
Haplotype networks of the member PLSs of PLC Clstr_599. (**A**) Member PLSs identified from isolates of American donors and Chinese donors. (**B**) Member PLSs identified from the metagenomic sequencing datasets of different environments according to the pairwise Mash distance (<0.01) with the member PLSs of Clstr_599. Circles represent different haplotypes, and the size is proportional to the number of PLSs belonging to the haplotype. Different colors denote the origins of the PLSs, and pie charts indicate the frequency of the PLSs with different origins within a haplotype. Transverse bars represent the number of different nucleotide bases between haplotypes. Black dots represent hypothetical haplotypes as intermedia of different existing haplotypes.

The haplotype network analysis of the 229 selected members of Clstr_599 derived from the isolates from Chinese and American donors resulted in 31 haplotypes (Figure 5A, Supplementary Table S11). The haplotypes Hap_10 and Hap_11 were predominant among these haplotypes according to the taxonomy of their hosts across 3 bacterial phyla and 4 phyla, respectively. Hap_9 and Hap_11 included the PLS members detected in the bacterial isolates from both American and Chinese donors. This suggested that the extensive exchange of the PLC Clstr_599 in various bacterial hosts across distinct bacterial phyla and environments occurred very rapidly. Assuming a mutation rate of ∼30 mutations per year per genome as *Helicobacter* pylori (genome size ∼1.67 M) (51,52), we estimated that the transmission of the Clstr_599 (genome size ∼5.6k) members that do not contain any mutations in various environments occurred 0–10 years ago. Several rare haplotypes (frequency <2%) likely had an independent (local) transmission and evolutionary history, e.g. the sublineage of Hap_1 to Hap_5 detected only in Chinese donors. Multiple haplotypes were found in the individual American_am, demonstrating the sequence diversity of the plasmid in one individual human gut. The haplotypes in Americam_am belonged to two distinct sublineages, one including Hap10 and Hap_11 and the other including Hap_16 to Hap_31. In addition, more than one prevalent haplotype that occurred in multiple human guts or even different populations, e.g. Hap_11 and Hap_25, existed in American_am, suggesting that Clstr_599 likely colonized the individual gut at least two times and highlighting the frequent transfer of Clstr_599 among the human populations.

We then constructed the haplotype network using the PLSs of Clstr_599 identified from the various environmental metagenomic datasets (Figure 5B, Supplementary Table S11), resulting in 55 haplotypes. EHap_16, EHap_50, EHap_55 and EHap_39 were distributed in different human populations or environmental niches, implying the rapid exchange of plasmids among these environments. This network also showed that the rare haplotypes in different niches from close geographic locations tended to have independent (local) transmission and evolutionary history. For example, EHap5, Ehap6, and Ehap_20 to Ehap_32 distributed in European people or European sewage, belonged to one sublineage.

Alignment of the replication initiation genes of Clstr_599 PLSs derived from the isolates showed that they were identical by 100%, suggesting that the replication mechanism is versatile for Clstr_599 in hosts across various genera. We then conducted haplotype analysis for the PLC Clstr_417 (Supplementary Figure S7 and Supplementary Table S11), which had the broadest host range (24 genera) among the identified PLCs and also is widespread in human populations and global sewage, resulting in patterns similar to those of Clstr_599. These patterns, again, suggest the very rapid exchange of plasmids across different environments.

## DISCUSSION

**Figure 6.**
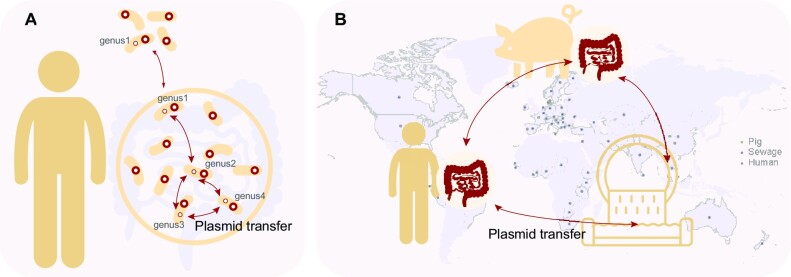
Putative transmission patterns of BHR plasmids derived from the human gut microbiome. (**A**) Cosmopolitan bacteria (e.g. genus 1) harboring the BHR plasmids came in and out of the human gut, and the plasmids were transmitted among different bacterial strains (*e.g*. genera 2, 3 and 4) within the gut microbiome, accompanied by accessory gene exchange. (**B**) Global transmission of the BHR plasmids likely occurred via human activities, thereby promoting gene exchange across different environments (e.g. the human population, livestock, and sewage water). The colored dots indicate the sampling locations of the metagenomic datasets used in this study.

Efforts to identify the plasmids enriched in the human gut and determine the host range of the plasmids are of interest to increase our understanding of the human gut microbiome. In this study, we report a large collection of information on human gut bacterial plasmids and their hosts. Our study identified the plasmids from the isolates generated by two large-scale gut bacterial culturing studies, and each of the bacterial isolates was plated into single colonies for culturing for at least two days before sequencing the genome (13,14). This ensured accurate host information for the human gut plasmids. We then identified a subset of PLCs with highly similar member PLSs that were shared among the hosts across phylogenetically distant bacterial taxa and geographically distant environmental niches. These BHR plasmids carrying various accessory genes were transmitted within the individual gut microbiome and were rapidly dispersed by some cosmopolitan bacteria, e.g. *E. coli*, in global human populations and various environmental niches, likely caused by increasing human contact in the era of industrialization, as indicated in Figure 6.

The Mash distance is roughly equivalent to 1-ANI (18). For bacteria, the intra-strain ANI is >99% (53), intra-species ANI is >95% (18,54) and inter-species ANI is <83% (55). We set a stringent similarity cutoff (pairwise 99% ANI) for clustering the PLSs into PLCs to avoid overestimating the host range of the PLCs. We also used pairwise Mash distances <0.05, <0.15 to group the PLSs, resulting in 2890, 1576 clusters, respectively (Supplementary Table S3). This further demonstrated the sequence diversity of PLCs we obtained. In particular, 3062 (57.0%) PLCs were detected with a minimum Mash distance >0.3 with the known plasmids. These potentially represented novel plasmid fragments (Supplementary Table S3). Besides, we compared the identified PLCs with the linear plasmids from the NCBI database (17.1%, 7502/43 857, https://ftp.ncbi.nlm.nih.gov/refseq/release/plasmid/, 2022/05/05) using Mash, resulting in 832 PLCs (including 2631 PLSs) having more than 85% ANI with those linear plasmids (Supplementary Table S12). This indicated that the linear plasmids also presented in the human gut and were included in our study.

The extent of BHR plasmids transferred among the complex bacterial community in the human gut has not been explored. By analysis of the NCBI 43587 plasmids (https://ftp.ncbi.nlm.nih.gov/refseq/release/plasmid/, 2022/05/05), we obtained 39623 PLCs (pairwise >99% ANI within a cluster), only 546 PLCs (1.4%) in the host cross genera (*n* = 3977 plasmid sequences, Supplementary Table S13). Of those, 3150 plasmid sequences (involved in 442 PLCs) were in the hosts of *Klebsiella*, *Enterobacter*, *Salmonella*, *Staphylococcus* and *Citrobacter*, which were mainly isolated from the clinical environments. Only 16 PLCs (*n* = 56 plasmid sequences, Supplementary Table S13) were discovered in bacterial hosts across distinct genera in the human or animal gut. Therefore, the data generated in this study greatly expanded the collection of BHR plasmids in the human gut microbiome.

Our study showed that BHR plasmids can widely spread accessory fitness genes for their bacterial hosts in various environments globally. Petersen, J. *et al.* reported that pLA6_12, a RepL-type plasmid, was detected in diverse members of the Roseobacter group in global marine environments (56), which is the only instance for global transmission of BHR plasmids (≥2 genera) with high similarity (>95%) to our knowledge. Here, we reported the widespread distribution of many BHR plasmids derived from the human gut (ANI > 99% for pairwise members for each plasmid) across a large geographic distance. This demonstrated that the rapid spreading globally of the BHR plasmids is common rather than an exception. In addition, the global transmission of such BHR plasmids was not limited to the hosts in the human gut but also in the animal gut and environmental sewages. Highly similar plasmids have been discovered to spread ARGs locally in a given environment, *e.g*. in a hospital (57). Here, we showed evidence of rapid global transmission of ARGs by hitchhiking widespread plasmids, highlighting the significance of monitoring such fast-spreading plasmids carrying ARGs or other interesting genes for human health.

In addition, the host information of the human gut plasmids also extended the previously published knowledge of the plasmids. For example, the plasmid pTRACA22 was obtained by TRACA, which missed the host information, while in our study, the hosts of the plasmid were *Lachnoclostridium* and *Subdoligranulum* because it was assigned to Clstr_2849 with a Mash distance < 0.01 (58,59). The plasmid Clstr_417 has been modified as a shuttle vector (pVAL-1, >99% identity) between *E. coli* and *Bacteroides* (60). Here, we determined Clstr_417 in hosts across 24 genera belonging to five phyla. This potentially extended the application range of pVAL-1 in the future.

In conclusion, our study reports a large number of human gut plasmids derived from the isolated bacterial draft genomes and shows that a subset of the plasmids has a broad host range, can persist in the human gut for a long duration and are widespread in the gut of global human populations and various environmental niches. The features of these plasmids suggest their great contributions to extensive HGT events, such as spreading ARGs, highlighting the potential implications of human plasmids for global human health. There are certain limitations in this study, e.g. the fragmentation of the draft genomes and the bias in the sampling of the isolates, leading to the fact that the plasmids we obtained are likely only part of the human gut plasmidome. Nevertheless, all these limitations cannot affect the conclusions we draw in this study. Our study will certainly advance our understanding of the human gut ecosystem.

## Supplementary Material

gkad498_Supplemental_Files

## Data Availability

The mentioned tools used for the data analysis were applied with default parameters unless specified otherwise. The related scripts and more datasets are available at https://figshare.com/s/cd21750d076848aca929 or https://github.com/SIAT-MaLab/Bacterial_plasmids.
