# Supplementary material for: Global transmission of broad-host-range plasmids derived from the human gut microbiome
Source: Nucleic Acids Res. 2023 Jun 7;51(15):8005–19. doi: 10.1093/nar/gkad498 (PMC10450197; doi:10.1093/nar/gkad498)
Supplement: gkad498_Supplemental_Files [file gkad498_supplemental_files.zip › SUPP_data-revised.pdf]

SUPPLEMENTARY TABLES

Table S1. The information of the isolates used in this study.

Table S2. The metagenomic samples used in this study.

Table S3. The information of the high-confidential PLSs identified in this study.

Table S4. The CGMs and PGMs.

Table S5. The validation of the workflow identifying PLSs.

Table S6. The benchmarking of the workflow identifying PLSs.

Table S7. The information of comPLCs.

Table S8. The PLCs classification including plasmid typing and network typing.

Table S9. The accessory genes identified in the PLSs.

Table S10. The prevalence of the PLCs in different environments.

Table S11. The results of haplotype analysis.

Table S12. The PLSs hitting to NCBI linear plasmids.

Table S13. The information of the NCBI cross-genera plasmid clusters.

SUPPLEMENTARY FIGURES

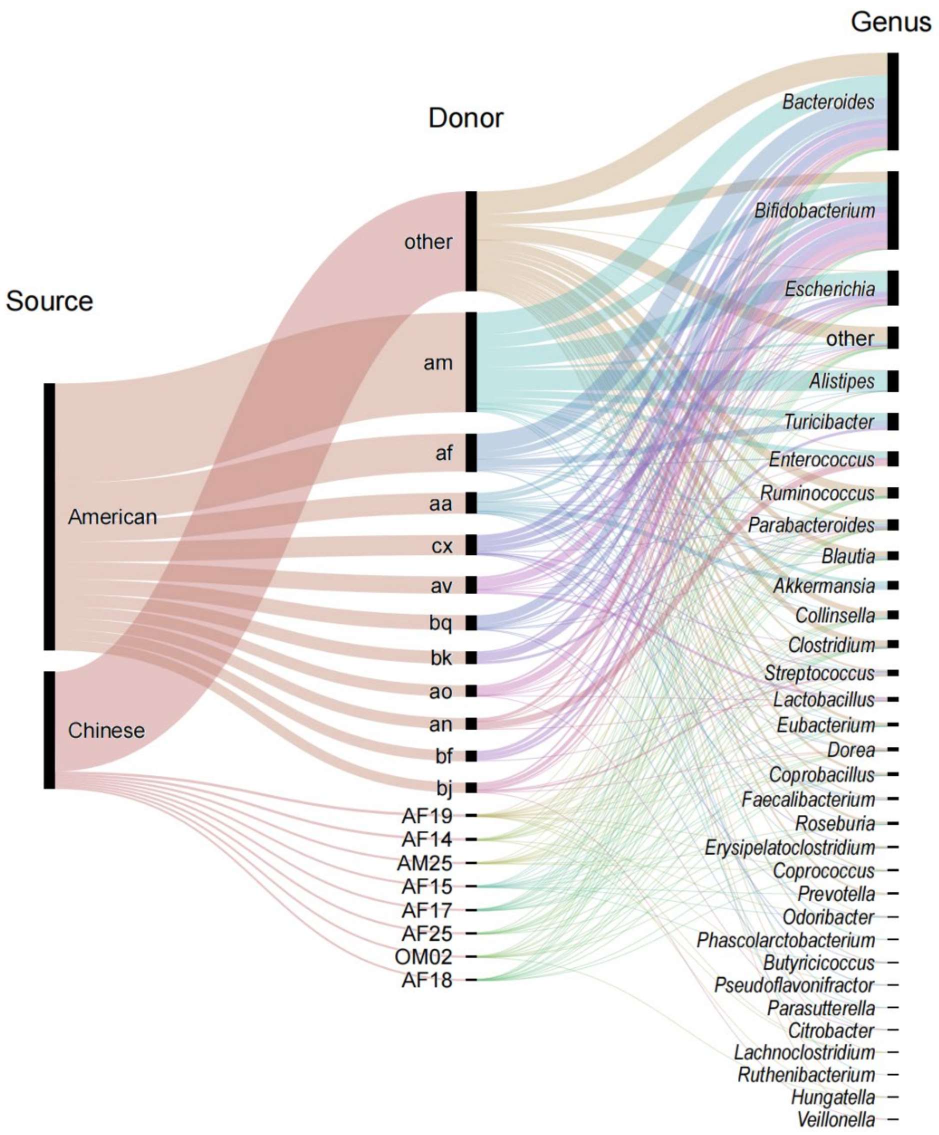

Figure S1. Information on the isolates collected from American and Chinese donors used in this study. The height of each bar is proportional to the number of isolates in the category. The “other” genera include genera with ≤ 10 isolates. The “other” donors include donors with < =25 isolates. The labels “am”, “af” and such of donors were the name of each individual person.

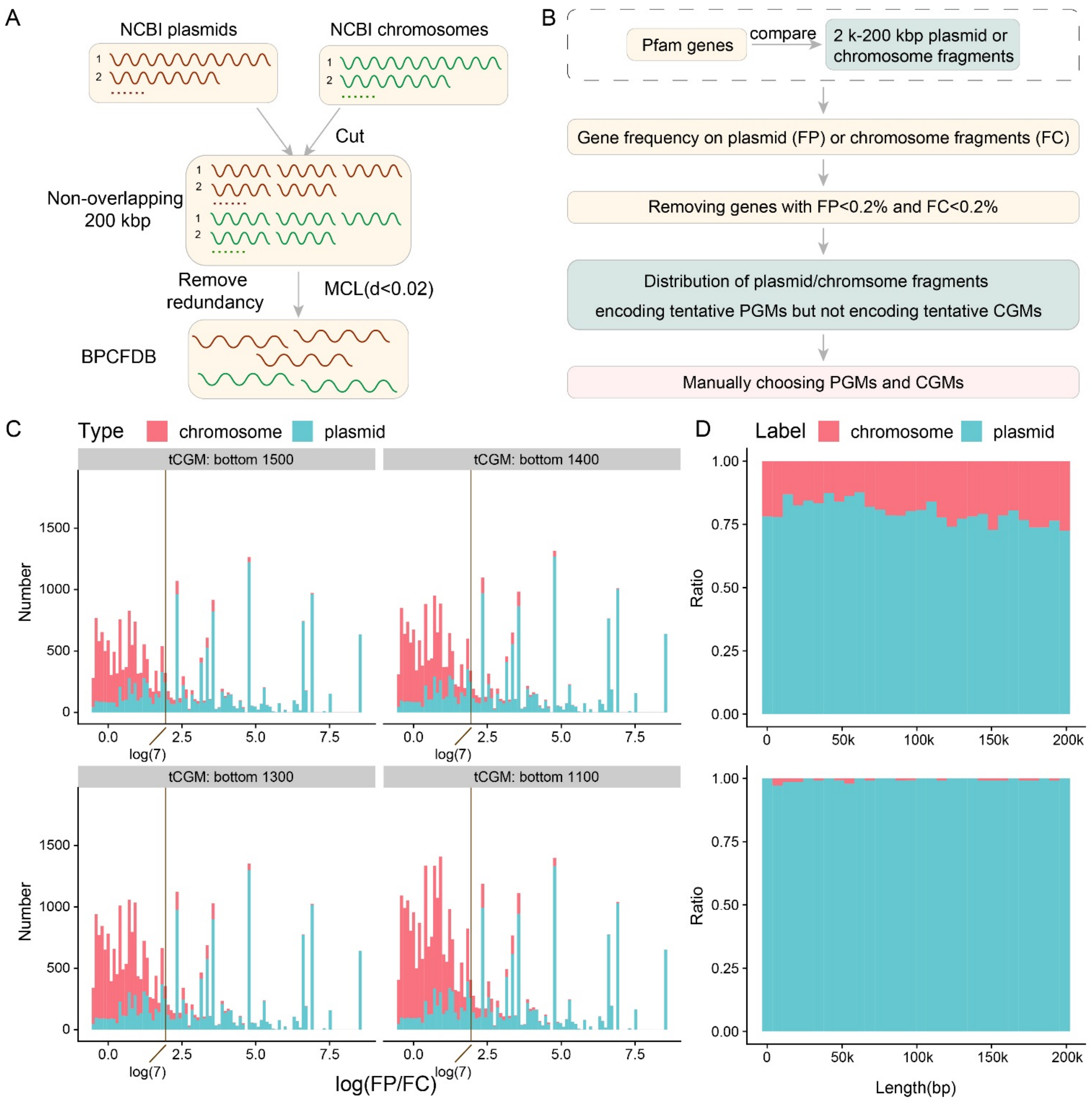

Figure S2. Identification of PLSs from human gut bacterial isolates. A. Overview of constructing BPCFDB (Bacterial Plasmid and Chromosome Fragment DataBase). More details are provided in the Methods. B. Overview of selecting plasmid-like gene markers (PGMs) and chromosome-like gene markers (CGMs). More details are provided in the Methods. C. Selection of PGMs and CGMs. The number of fragments encoding tentative PGMs (genes with the top 1000 FP/FC values) along FP/FC values is shown. Each fragment was counted only once for its encoding gene with the largest FP/FC value. The fragments encoding tentative CGMs (tCGMs, genes with bottom 1500, 1400, 1300, and 1100 FP/FC values) were removed in different panels. D. Validation of the identification workflow along the different lengths. The top is the recall distribution. The color shows the predicted origin (plasmid/chromosome) of all mimic

plasmid contigs. The bottom is the precision distribution. The color shows the real origin (plasmid/chromosome) of predicted PLSs.

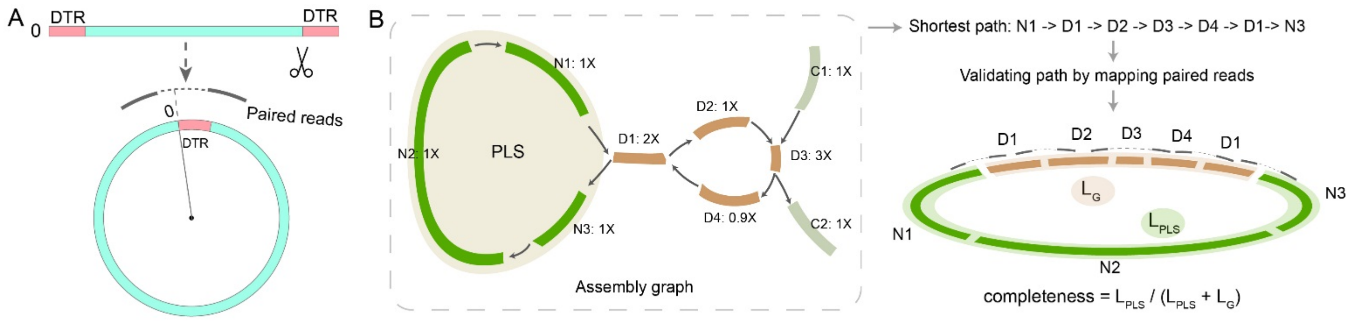

Figure S3. Estimating the completeness of PLCs. A. Detection of PLSs with ~100% completeness. DTRs, direct terminal repeats. B. Estimation of the completeness of PLSs. This was only applicable to those PLSs that each was

inferred in a circular path in an assembly graph (see Methods). The shortest path for a PLS in its corresponding genomic assembly graph, like N1→D1 → D2 → D3 → D4 → D1 → N3, was determined by python package NetworkX. The path was checked whether all adjoining nodes were mapped by paired sequenced reads for validation. For a node with multiple copy numbers like D3, only when there were paired reads mapped to its upstream (D2) and downstream (D4) adjoining nodes, it was not considered as the signal for skipping integrative elements and taken for further analysis. The L_{PLS} was the length of PLS. The L_G was the sum of length of all nodes on the shortest path excluding the beginning and ending nodes (D1, D2, D3, D4, D1).

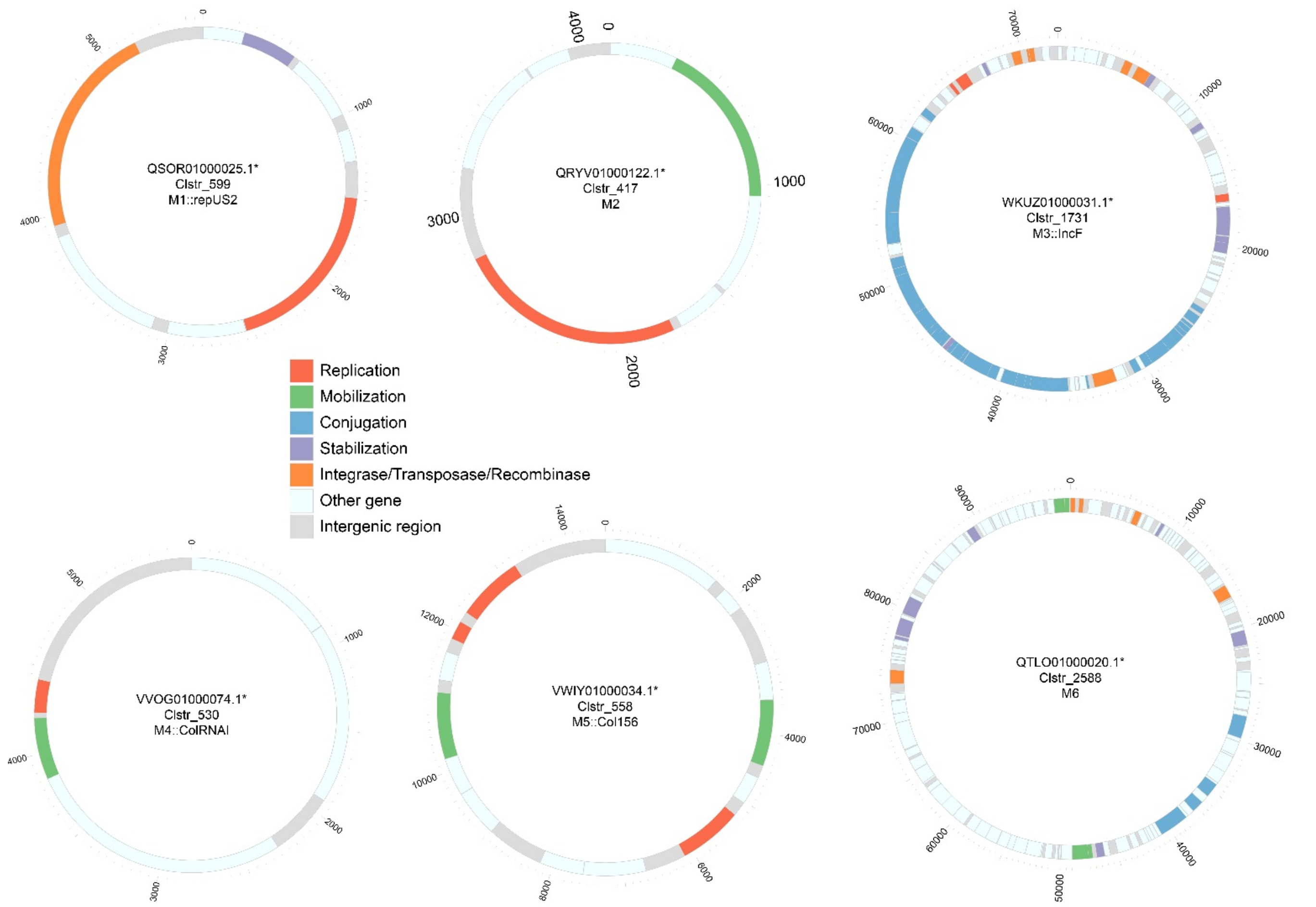

Figure S4. Structures of representative PLSs of M1-M6 NTGs. Genes with various functions, including plasmid replication, mobilization, and conjugation, stabilization and transposase/recombinase/integrase activities, are colored. Texts within each circle show the PLS contig ID(*), PLC ID and NTG to which it belongs. For each sequence, overlapping ends were removed, and beginning points were reselected to avoid splitting a gene on two ends.

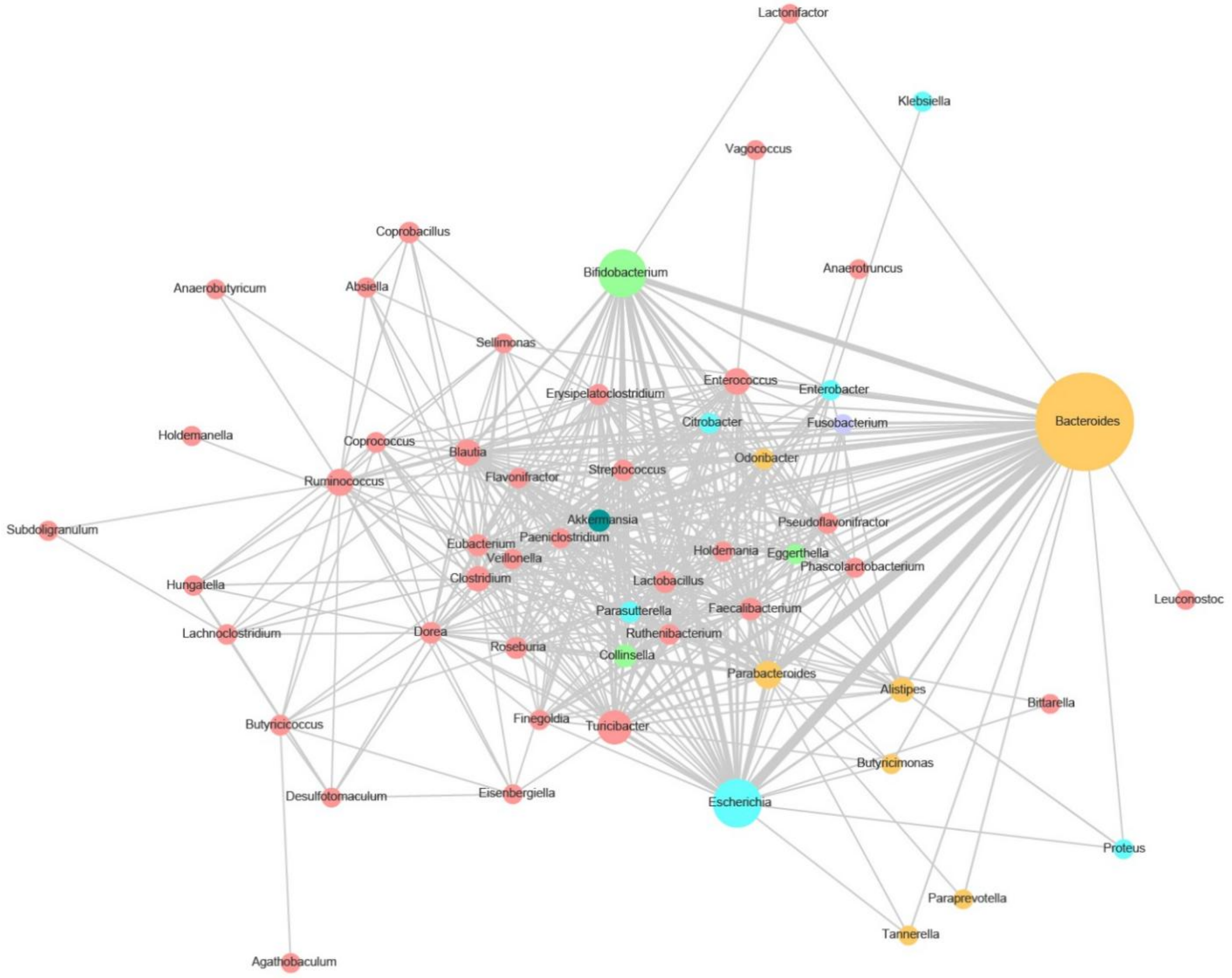

Figure S5. Network of the isolates connected by the shared PLCs. Each node represents a genus. The colors show different phyla. The size of the nodes is proportional to the number of isolates belonging to that genus. Two genera are linked if they share the same PLCs. The width of the edges is proportional to the number of shared PLCs.

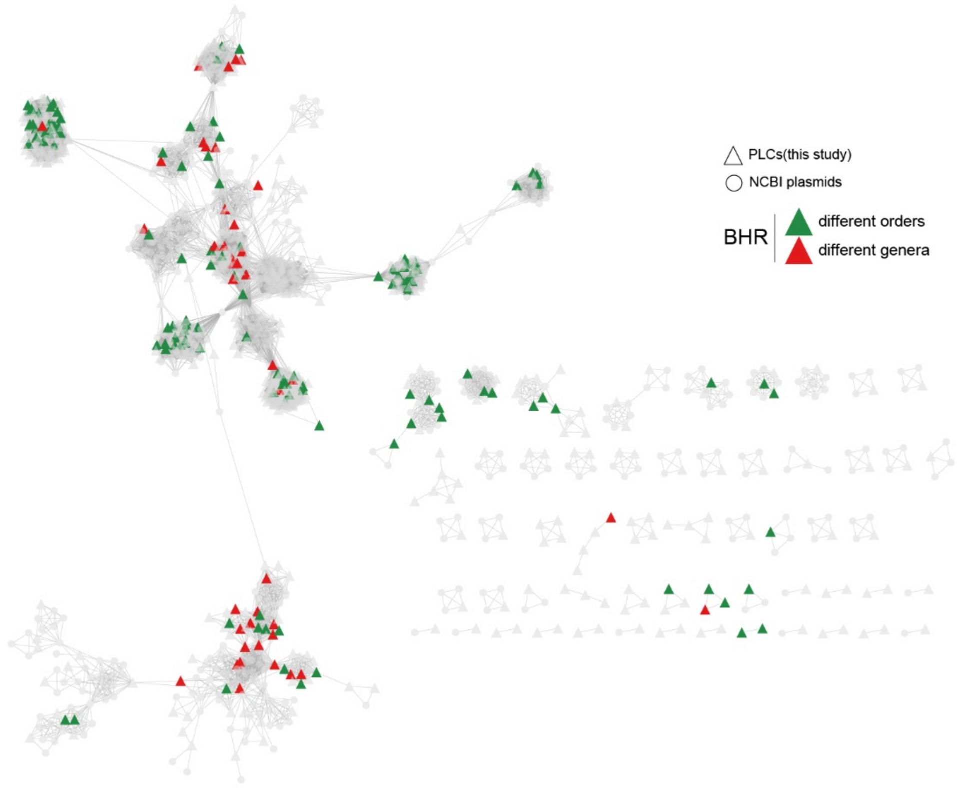

Figure S6. The diversity of BHR comPLCs in the human gut. Cross-genera and cross-orders comPLCs were colored.

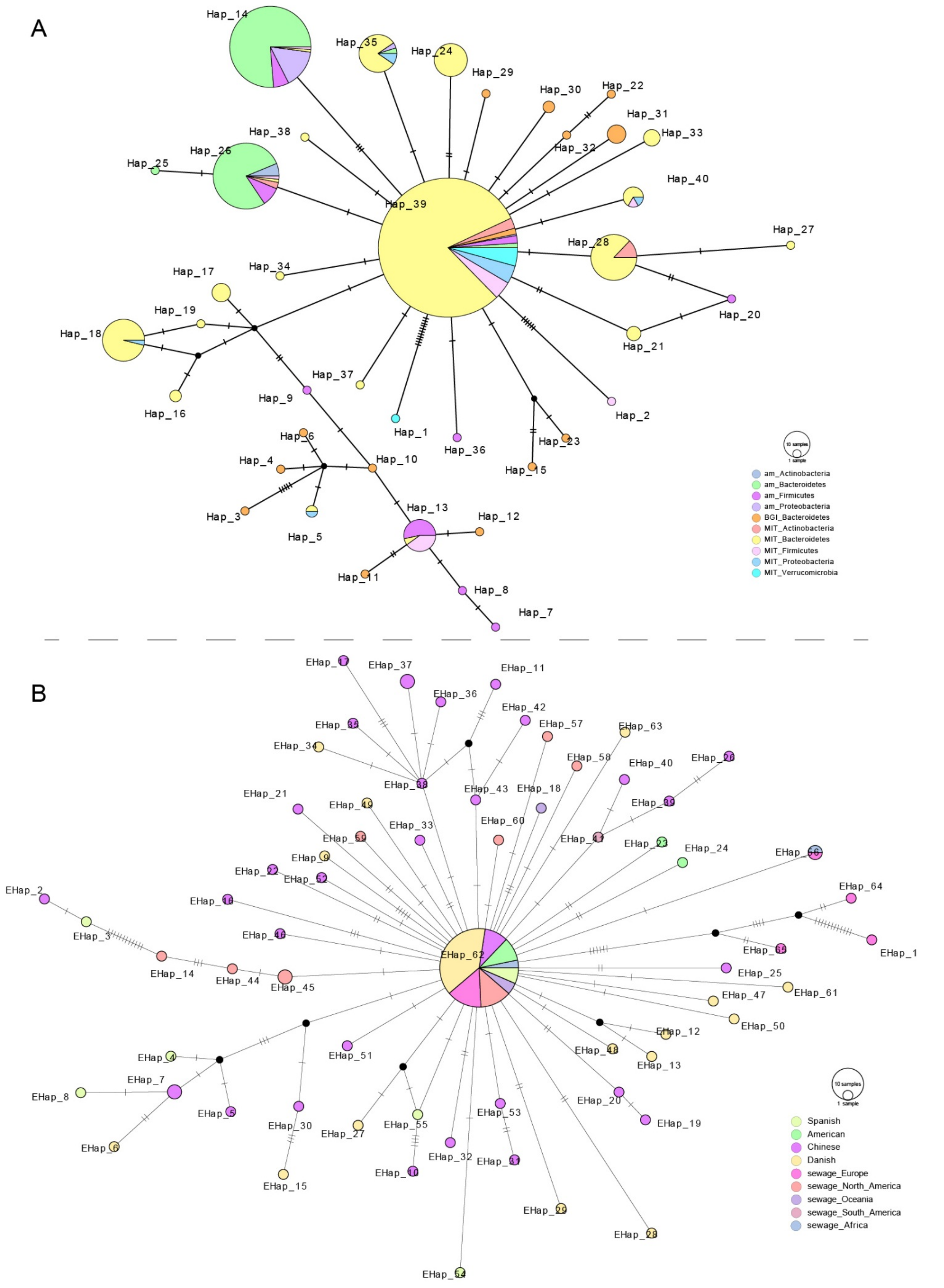

Figure S7. Haplotype networks of Clstr_417 with member PLSs identified from isolates of American and Chinese donors (A) and different environments (B). A. Member PLSs were identified from isolates of American donors and Chinese

donors. B. The PLSs were identified from the metagenomic sequencing datasets of different environments according to the pairwise Mash distance (0.01) with the member PLSs of the cluster Clstr_417. Circles represent different haplotypes, and the size is proportional to the number of PLSs belonging to the haplotype. Different colors denote the origin of the PLSs, and pie charts indicate the frequency of the PLSs with different origins within a haplotype. Transverse bars represent mutational steps between haplotypes. There was a reverse complement mutation (10 bp) in the intergenic region between the rep gene and chm2bp gene. This was seen as a single base mutation in the analysis because one mutation could cause such a complement mutation.
